# Experimental measurements of the permeability characteristics of rare earth ore under the hydro-chemical coupling effect

**DOI:** 10.1039/c8ra00446c

**Published:** 2018-03-26

**Authors:** Xiaojun Wang, Yulong Zhuo, Kui Zhao, Wen Zhong

**Affiliations:** Jiangxi Key Laboratory of Mining Engineering, Jiangxi University of Science and Technology Jiangxi 341000 China; School of Resources and Environment Engineering, Jiangxi University of Science and Technology Jiangxi 341000 China yglmf_zk@163.com +86-139-7078-6532

## Abstract

Two coupling processes—solution seepage and chemical replacement—occur in the *in situ* leaching process of ion-absorbed-rare-earth ore. In this study, saturated leaching tests were applied to investigate the permeability characteristics of rare earth ore under the hydro-chemical coupling effect. The pore radius distributions based on two leaching solutions (H_2_O and (NH_4_)_2_SO_4_) were obtained by nuclear magnetic resonance detection technology. The results indicated that 10–25 μm and 4–10 μm pores are dominant in the ore under solution leaching using H_2_O and (NH_4_)_2_SO_4_, respectively. A “black belt” in the pores of (NH_4_)_2_SO_4_ leaching was discovered from the reconstruction of the pore structure distribution image. The results also reveal that the hydraulic conductivity will initially increase, then decrease and finally increase during the leaching process. The hydro-chemical coupling effect will lead to variations in the pore structure and permeability of rare earth ore. The pore radius will increase due to solution seepage, whereas it decreases due to the particle recombination induced by chemical replacement. The permeability of rare earth ore is influenced more by chemical replacement than by solution seepage.

## Abstract 2

Two coupling processes—solution seepage and chemical replacement—occur in the *in situ* leaching process of ion-absorbed-rare-earth ore.

## Graphical abstract

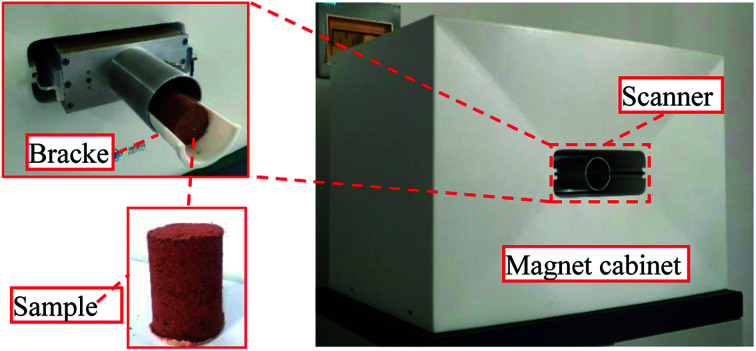

## 1. Introduction

Ion adsorption-type rare earth ore is one of the most precious and rare types of ore in the world and is found in intensely weathered granites in shallow ground.^1–5^ The *in situ* leaching mining method is a mature mining technology and utilizes the chemical replacement reaction of the leaching solution in the process of ore body seepage to recover rare earth cations, thus enabling the extraction of rare earth elements.^6–9^ The permeability characteristics of the leaching solution in the ore body rely heavily on the distribution of the internal microscopic pore structure. The morphology and distribution of the microstructure are influenced by both solution seepage and chemical action.^10,11^ Thus, measurement of the permeability characteristics of rare earth ore under the hydro-chemical coupling effect is important when investigating the mechanisms of leaching and penetration.

The matrix of rare earth ore is intensely weathered granites, which belongs to the same discrete heterogeneous body as ordinary rock and soil media. Several researchers have recently studied the hydraulic characteristics of rock and soil using different methods. These methods have been improved significantly due to advancements in technology (*e.g.*, computed tomography (CT) and nuclear magnetic resonance (NMR) detection technology).^12–14^ Kong *et al.*^15,16^ found that the hydraulic conductivity of sand and gravel soil increases with an increasing percentage of fine particles. Fan *et al.*^17^ observed a quadratic polynomial relationship between the hydraulic conductivity and effective grain size in a laboratory test of sand gravel. Santoso *et al.*^18^ investigated the spatial variability of the saturated hydraulic conductivity of soil and then used this variability to estimate the probability of slope failure. Nosrati *et al.*^19^ applied micro-CT to scan the internal structure of nickel granulation ore and established the microstructure distribution law. Yan Xiaoqing *et al.*^20^ introduced a micro-parameter to describe the pore characteristics of soil. Li *et al.*^21^ considered the relationship among the pore structure, void ratio and water content in the soil drying process.

The *in situ* leaching of ion-type rare earth involves the two important processes of ion exchange and solution infiltration. Ion exchange is a chemical replacement process, whereas solution infiltration is a physical migration process. However, few studies have focused on the variations in the permeability of rare earth ore induced by these two processes. The main objective of this study was to investigate the influence of the hydro-chemical coupling effect on the permeability of rare earth ore based on the evolution mechanism of the microscopic pore structure obtained by NMR detection technology.

## 2. Experimental

### 2.1. Principle of the experiment

#### 2.1.1. Chemical replacement

Rare earth (RE) elements are found in strong weathered granite in the form of ions. An (NH_4_)_2_SO_4_ solution is typically used in the leaching process.^22,23^ The rare earth cations absorbed on the ore body will be replaced in the leaching solution after reacting with NH_4_^+^, which has more active chemical properties, as shown in formula (1). This strong chemical reaction occurs throughout the leaching process. The ion exchange involves the adjustment and reconstruction of the mineral microstructure, leading to changes in the crystal structure chain. The seepage effect of the leaching solution also impacts the microstructure of the ore body.

1 2(Kaolin)^3−^·RE^3+^ + 3(NH^+^_4_)_2_·SO^2−^_4_ = 2(Kaolin)^3−^·(NH^+^_4_)_3_ + RE^3+^_2_(SO^2−^_4_)_3_

#### 2.1.2. Microstructure tests

NMR, a new type of detection technology, has been widely applied in the field of geotechnical engineering.^24^ The most important characteristics of this technology are that it can rapidly, accurately and quantitatively measure the microstructure porosity and pore size distribution while not destroying the structure of the rock and soil. Therefore, this is the most suitable detection technology for the analysis of the microstructure evolution of a rare earth ore body during the leaching process.

**Fig. 1:**
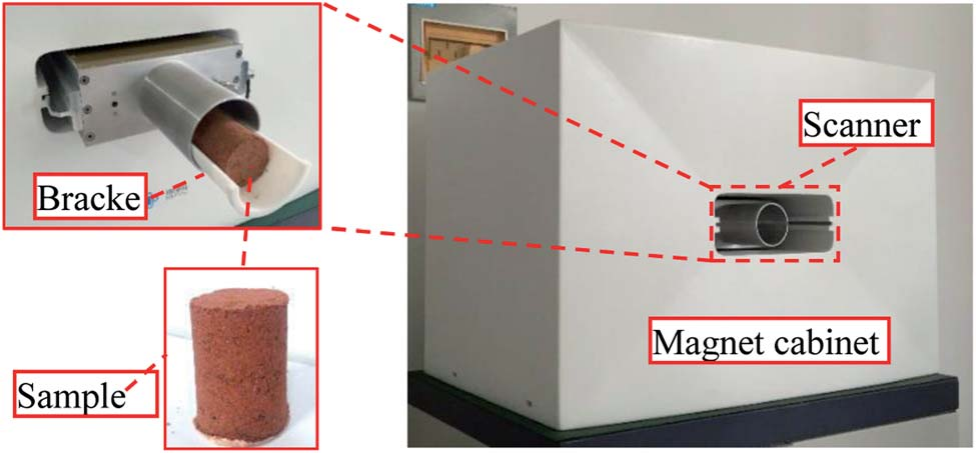
PQ-OO1-type Mini-NMR.

The micropore structure of the sample was measured using a PQ-OO1 type Mini-NMR (Suzhou Niumag Analytical Instrument Corporation, Suzhou, China), as shown in Fig. 1. Permanent magnets possess a magnetic field intensity of 0.52 T. The valid experiment area of the sample is *ϕ*60 mm × 60 mm in the experimental process, and the temperature of the permanent magnets should be 32 ± 0.01 °C, ensuring the stability and uniformity of the experimental magnetic field. Two typical samples are acquired before leaching and two samples are acquired after leaching to analyze and detect the micropore structure every hour based on the leaching interval. After the detection, leaching is performed on the same sample, and the consistency of the sample when conducting the microstructure detection must be ensured to analyze the evolution of microstructures from the leaching process on the rare earth ore body.

#### 2.1.3. Hydraulic conductivity test

The solution will seep into the pores of the ore during the leaching process. The hydraulic conductivity can be calculated based on the following relationship between the flow rate and hydraulic gradient proposed by Darcy:

2 *Q* = *kiA*

3 *v* = *ki*

where *Q* is the flow rate (cm^3^ s^−1^), *k* is the hydraulic conductivity (cm s^−1^), *i* is the hydraulic gradient (%), *A* is the sectional area (cm^2^) and *v* is the seepage velocity (cm s^−1^).

**Fig. 2:**
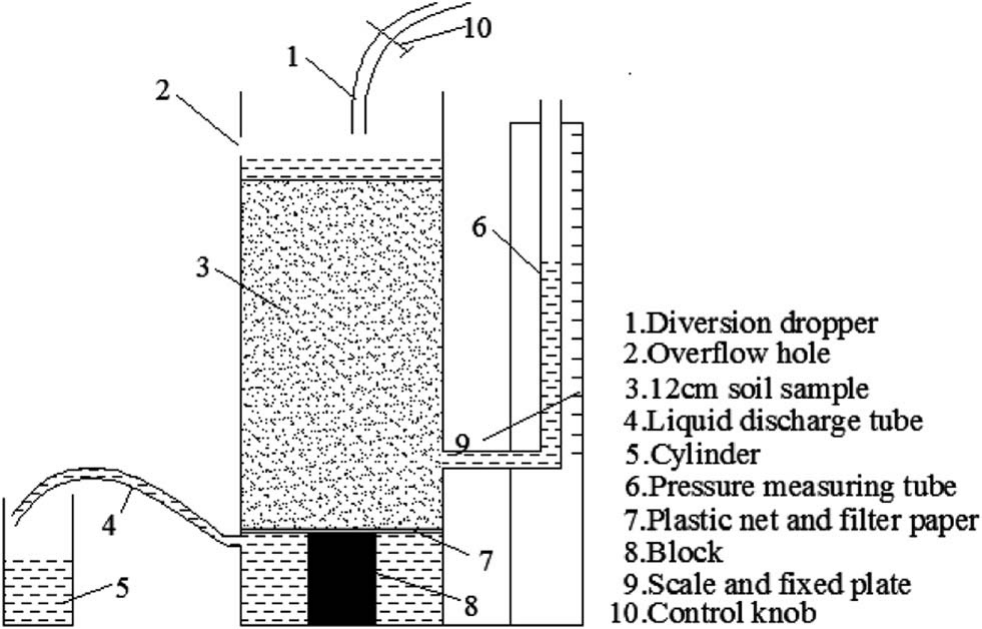
Schematic of the hydraulic conductivity test system during leaching.

However, this calculation method may not be suitable for calculating the hydraulic conductivity of rare earth ore because the hydraulic conductivity will change constantly during the leaching process. Therefore, we designed and developed an instrument and method that can be applied to calculate the hydraulic conductivity of ion-absorbed rare earth ore during the leaching process, as shown in Fig. 2. The equation can be deduced from Darcy's law as follows:

4 *k* = (*QL*)/(*A*Δ*h*)

where *k* is the hydraulic conductivity (cm s^−1^), *Q* is the flow rate (cm^3^ s^−1^), *L* is the height of the specimen (cm), *A* is the sectional area (cm^2^), Δ*h* is the head difference between the tube and injection water level (cm).

When we performed this test, the rare earth ore specimen was set in the container and the control button was opened. Moreover, the drainage tube should remain open to ensure seepage of the solution. Δ*h* and *Q* were recorded hourly such that the hydraulic conductivity could be calculated using eqn (4).

### 2.2. Experimental method and process

Rare earth samples were taken from a rare earth mine in southern China Longnan County. The grade of Longnan ionic rare earth ore is low and mainly in the range of a few thousandths. However, rare earth elements with complete distributions, especially heavy rare earth elements, are abundant. The content of radioactive elements is low, and most ore deposits are non-radioactive. The rare earth elements are mainly adsorbed on the surface of clay minerals in ionic form in fully weathered layers.

**Fig. 3:**
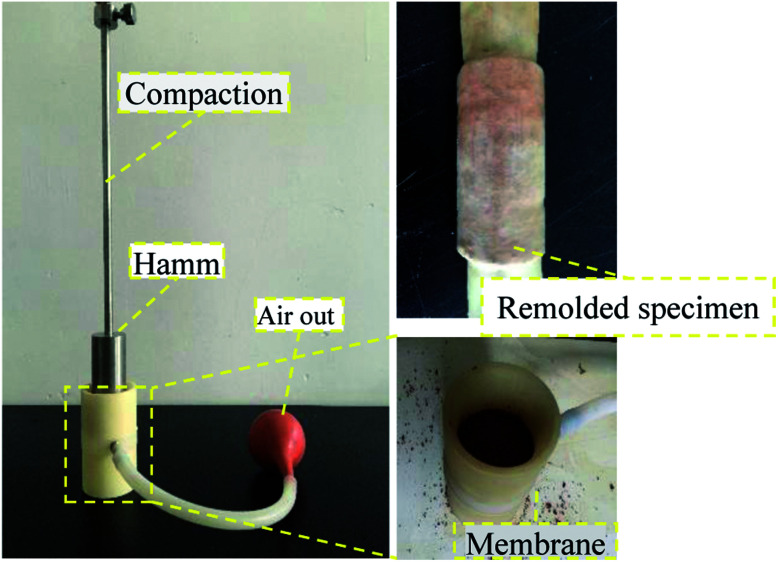
Manufacturing process of the remolded soil specimens.

The sample was acquired by collecting rare earth ore from the mine, and the sample size coincided with the effective detection area of the NMR. The diameter-to-height ratio was 40 mm : 60 mm. The soil samples were air dried before the test, and the water content was 0%. During the remodeling process, the density and moisture content of the original soil sample were maintained to the maximum extent possible, as shown in Fig. 3. Each sample was pre-tested by comparing densities to determine the appropriate number of compaction times. This number was determined to be 3, and the compaction height was 30 cm. A standard compaction device with a compaction hammer diameter of 40 mm was selected. After phase detection, the rare earth content (REO) of the original sample was 0.065 ± 0.003%, which conformed to the experimental requirements. The physical parameters of the remodeled rare earth sample are provided in Table 1.

**Table 1:** Physical parameters of the rare earth sample

Parameter type	Geometry dimension/mm		Density, g cm^−3^	Moisture content, %
	Diameter	Height		
Value	40	60	1.75	15

**Fig. 4:**
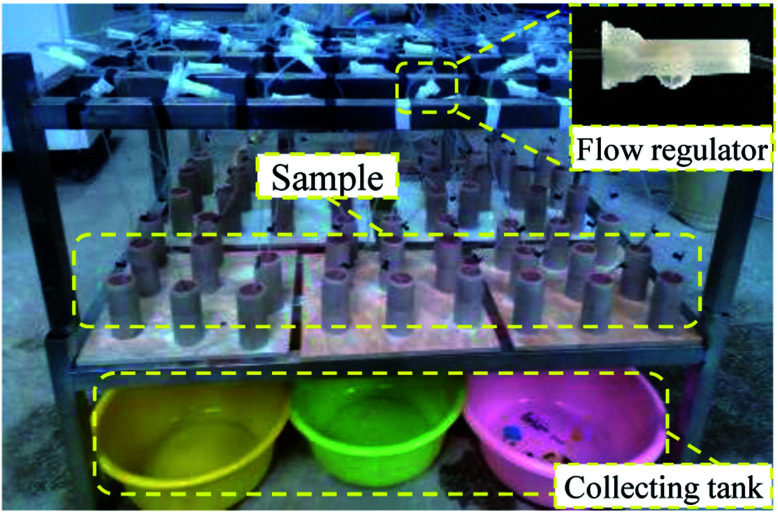
Simulation of the leaching.

A contrastive analysis experiment was designed to analyze the evolution law of the pore structure of a rare earth sample during the ion exchange process. The leaching experiment of remodeling a rare earth sample was conducted on the simulated leaching experiment table, as shown in Fig. 4. The leaching experiment is divided into two parts. In the first part, the leaching duration is 6 h, and pure water (H_2_O) is selected as the leaching fluid. The chemical replacement reaction is not involved in this process. In the second part, the leaching duration is still 6 h, but (NH_4_)_2_SO_4_ solution, which is commonly used in industrial experiments, is selected as the leaching fluid (concentration of 2.5%). Because rare earth ore is a natural acid–base buffer, the solution pH varies from 2–10, and the leaching rate is not affected. In the experiment, the pH of the ammonium sulfate solution was adjusted to approach that of water (pH = 6.5) to avoid the impact of the pH on the test results. According to the replacement principle, the second part is the ion exchange process. The experimental results of these two parts are analyzed.

**Fig. 5:**
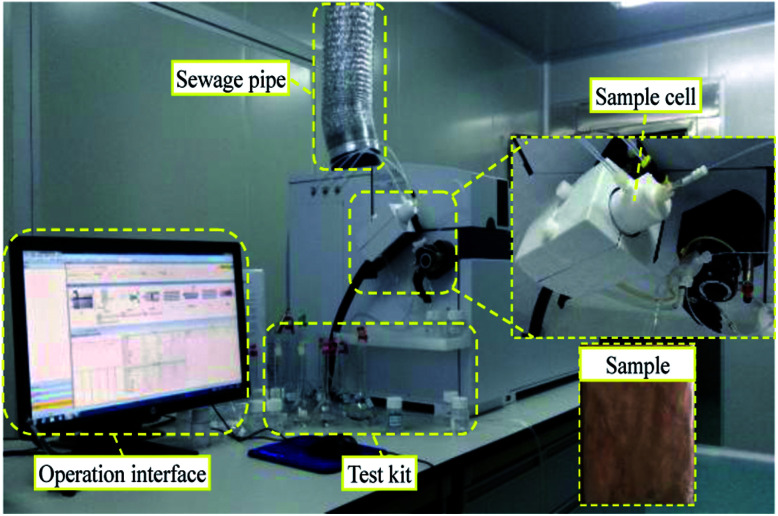
Agilent 8800 plasma mass spectrometer.

The experiment is divided into 1 h intervals to effectively identify the ion exchange rate of the mineral leaching chemical replacement process in different phases. The REO phase detection was conducted on the sample after each hour of leaching. When performing the detection, two complete samples are taken and then dried. Then, the samples are powdered. The REO of the sample is obtained using an Agilent 8800 plasma mass spectrometer, as shown in Fig. 5, and these results can represent the mineral leaching ion exchange process.

## 3. Analysis of the experimental results

### 3.1. Change in the REO during the leaching process

When leaching begins, detection is performed on the rare earth sample each hour to acquire the REO in different periods to analyze each phase of the ion exchange reaction during the leaching process. The experimental results of each period are shown in Table 2.

**Table 2:** REO in the leaching process

Leaching solution	Leaching time/h	REO/10^−6^ g t^−1^	Leaching solution	Leaching time/h	REO/10^−6^ g t^−1^
H_2_O	1	652	(NH_4_)_2_SO_4_ (2.5%)	7	630
	2	647		8	600
	3	657		9	470
	4	641		10	290
	5	639		11	200
	6	650		12	180
				13	179
				14	180

**Fig. 6:**
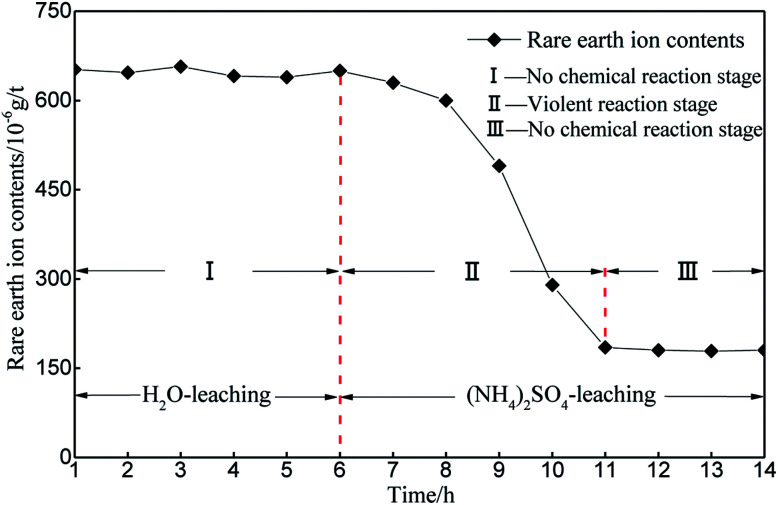
REO changing curve during the leaching experiment process.

Considering the REO trend curve obtained for the leaching process in Fig. 6, H_2_O is taken as the leaching solution in the first phase. The change in the REO of the sample during the 6 h of leaching is not obvious. A slight change can be observed in Table 2, which is due to the inconsistency between the samples (the initial REOs of the samples are similar, but differences exist among the individual samples). Thus, the ion exchange reaction, as is shown in formula (1), does not occur inside the sample, and the rare earth cations do not ooze out with the leaching solution when applying H_2_O as the leaching solution. After the first 6 h, the leaching solution is replaced by (NH_4_)_2_SO_4_ with a concentration of 2.5%. In the subsequent detection, the REO level gradually declines in the sample. The REO declines gradually between the 6^th^ and 8^th^ hours, mainly because the leaching solution containing rare earth cations do not ooze out in large amounts but instead remain in the sample matrix. Over time, the chemical replacement reaction occurs intensively in the sample matrix, and the leaching solution containing rare earth cations continuously ooze out. Fig. 6 illustrates that the REO in the sample matrix decreases dramatically between the 8^th^ and 10^th^ hours. Between the 10^th^ and 12^th^ hours, the REO drops to below 200 and the change begins to flatten, demonstrating that the ion exchange reaction is nearly complete in the sample after the 10^th^ hour. The above analysis demonstrates that the phase during the first 6^th^ hours does not have a chemical replacement reaction, the phase between the 6^th^ and 11^th^ hours involves intensive ion exchange and the ion exchange reaction is completed after the 12^th^ hour.

### 3.2. Pore radius distribution during leaching

The variation in microscopic pore structure induced by seepage and the coupling effect of seepage and chemistry can be reflected by the distribution of different pore radii.

The entire leaching process was divided into three phases: in the first phase (1–6 h), the specimen was leached only by H_2_O, and only seepage exited. Both H_2_O and ammonium sulfate ((NH_4_)_2_SO_4_) are used in the second phase (6–7 h); therefore, both seepage and a chemical reaction occurred within the specimen. In the last phase (7–14 h), the specimen was leached only by (NH_4_)_2_SO_4_. Both seepage and the chemical reaction occurred from the 7^th^ to 11^th^ hours, and only seepage occurred after 11 hours.

**Fig. 7:**
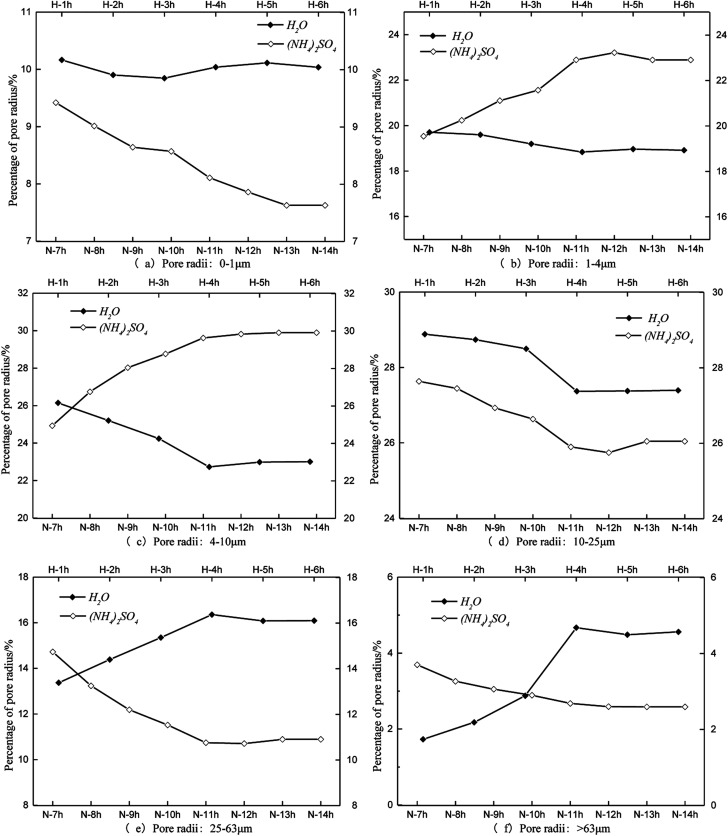
Results of the pore radius distributions.

The same pore radius distribution during the leaching process of the specimens is presented in Fig. 7. The percentage of pore radii between 0 and 1 μm remained steady at approximately 10% under the condition of water seepage (Fig. 7a). The percentage of pore radii between 0 and 1 μm decreased during the leaching process with (NH_4_)_2_SO_4_. The percentages of pore radii between 1 and 4 μm and between 4 and 10 μm decreased over time when leached by H_2_O, whereas they increased when leached by (NH_4_)_2_SO_4_ (Fig. 7b and c, respectively). The percentage of pore radii between 10 and 25 μm decreased when leached both by H_2_O and (NH_4_)_2_SO_4_ (Fig. 7d). The percentage of pore radii larger than 25 μm increased substantially when leached by H_2_O and decreased substantially when leached by (NH_4_)_2_SO_4_ (Fig. 7e and f, respectively).

**Fig. 8:**
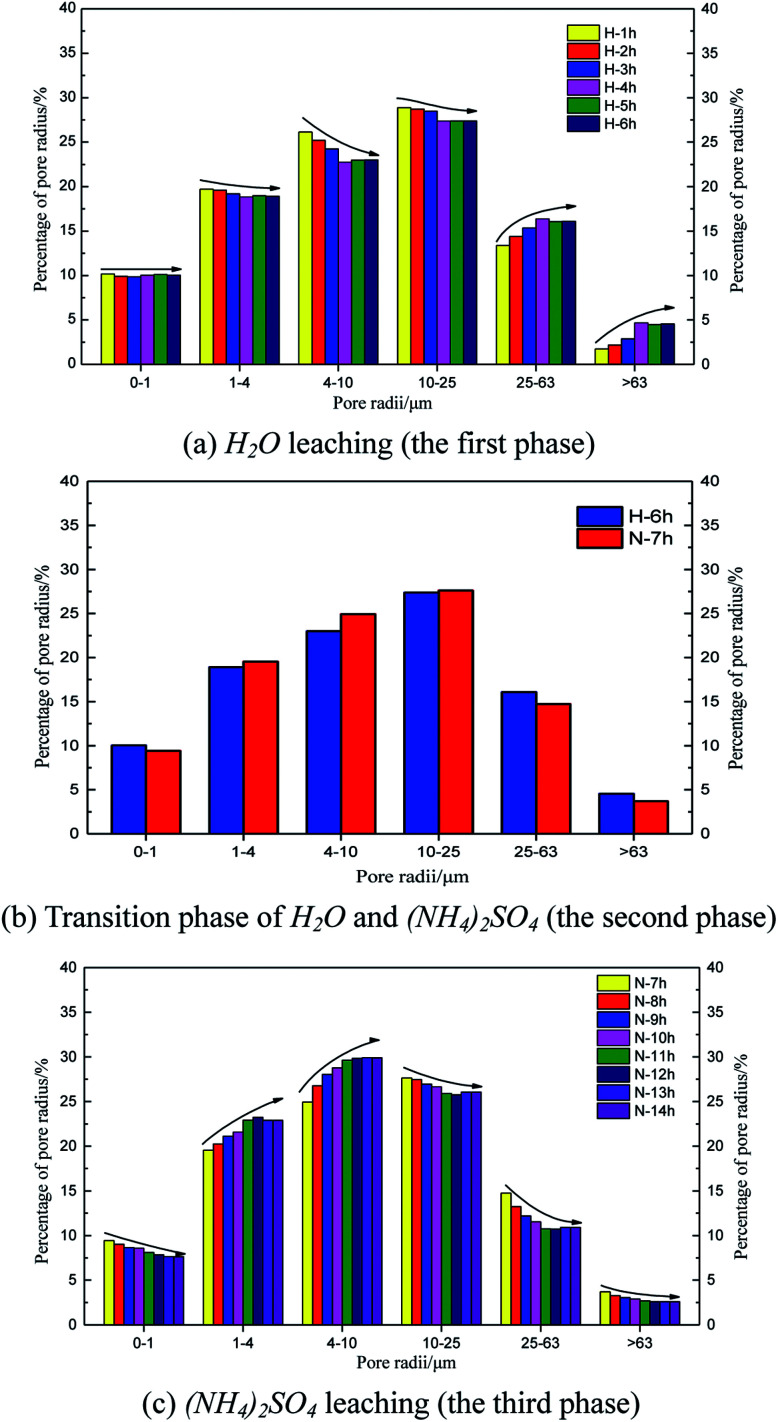
Results of the pore radius distributions during the leaching process.

Fig. 8 shows the time histories of the pore radius. The main pore radius size was a medium pore for the H_2_O leaching condition (Fig. 8a). The largest percentage of radii were between 10 and 25 μm, followed by those between 4 and 10 μm. Considering the curves obtained for each hour, the proportion of the pore radii between 10 and 25 μm and between 4 and 10 μm decreased over time; however, the proportion of pore radii between 25 and 63 μm and larger than 63 μm increased over time. These results demonstrate that the pore radius of the rare earth ore would increase under water seepage. Fig. 8c and d indicate that when the proportion of medium or large pores changed from 1 to 4 h and stabilized from 4 to 6 h, the effect of seepage on the pore structure decreased over time. At this time, after we changed the solution from H_2_O to (NH_4_)_2_SO_4_, the pore structure changed considerably again 1 h later (Fig. 8b). The proportions of pore radii between 1 and 4 μm, 4 and 10 μm, and 10 and 25 μm began to increase, whereas proportions of pore radii between 25 and 63 μm and larger than 63 μm decreased. Fig. 7 indicates that in contrast with samples leached by H_2_O, the proportion of large pores would decrease and the proportion of medium pores would increase when the pore structure is leached by (NH_4_)_2_SO_4_. In this state, pore radii between 4 and 10 μm are most common. Thus, the chemical replacement of (NH_4_)_2_SO_4_ plays an important role in the permeability of ore due to its compressed structure.

### 3.3. Imaging analysis of the microstructure evolution

**Fig. 9:**
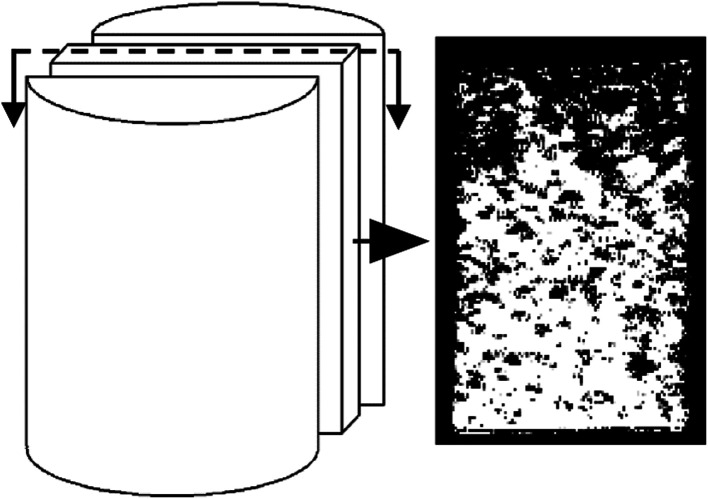
Typical cutting plane.

The analysis in Section 3.2 demonstrated that the ion exchange reaction between the rare earth ore body and leaching solution changes the structural chain among particles, leading to the recombination of particles among inner pores, and gives rise to new changes in the micropore structure. The reconstruction imaging technology NMR is applied to conduct reconstruct images of the scanning data at regular intervals, yielding an inversion image of the microstructure. The inversion image is a projected display in the form of two-dimensional sectioning used to intuitively analyze the micropore structure change law. The leaching solution infiltrates along the axis of the sample, and thus, the typical cutting plane is selected in the center of the sample along the generatrix, as shown in Fig. 9.

**Fig. 10:**
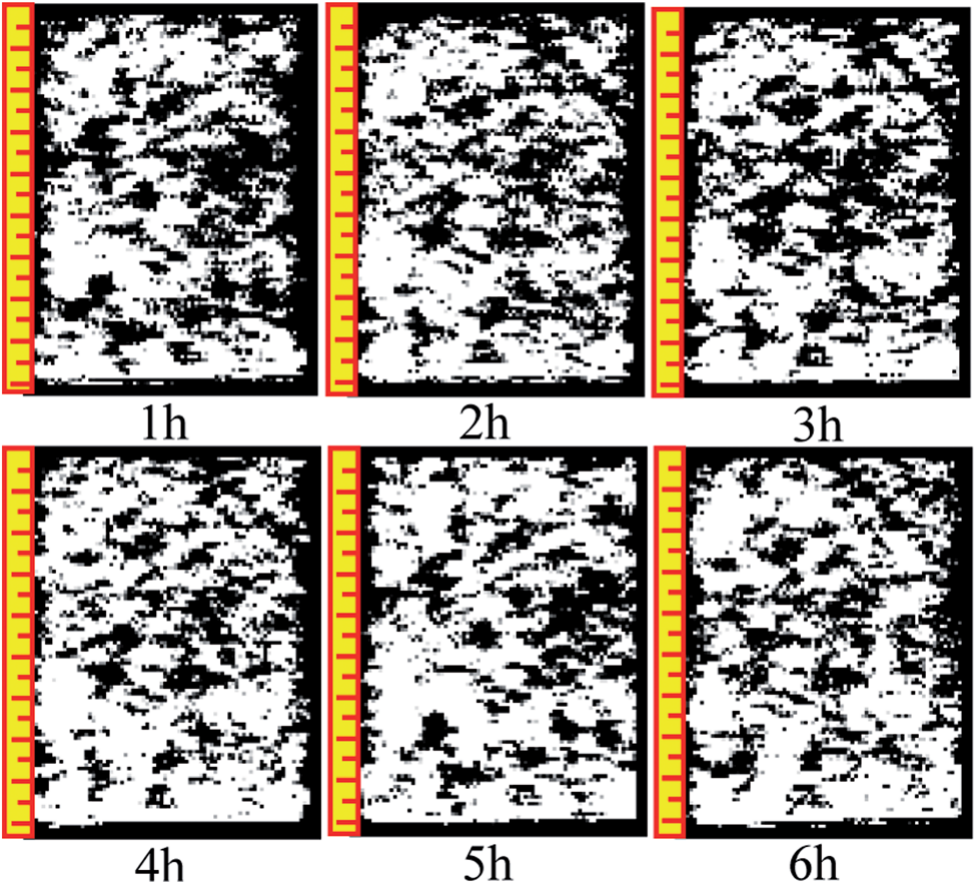
Phase of pure H_2_O leaching.

**Fig. 11:**
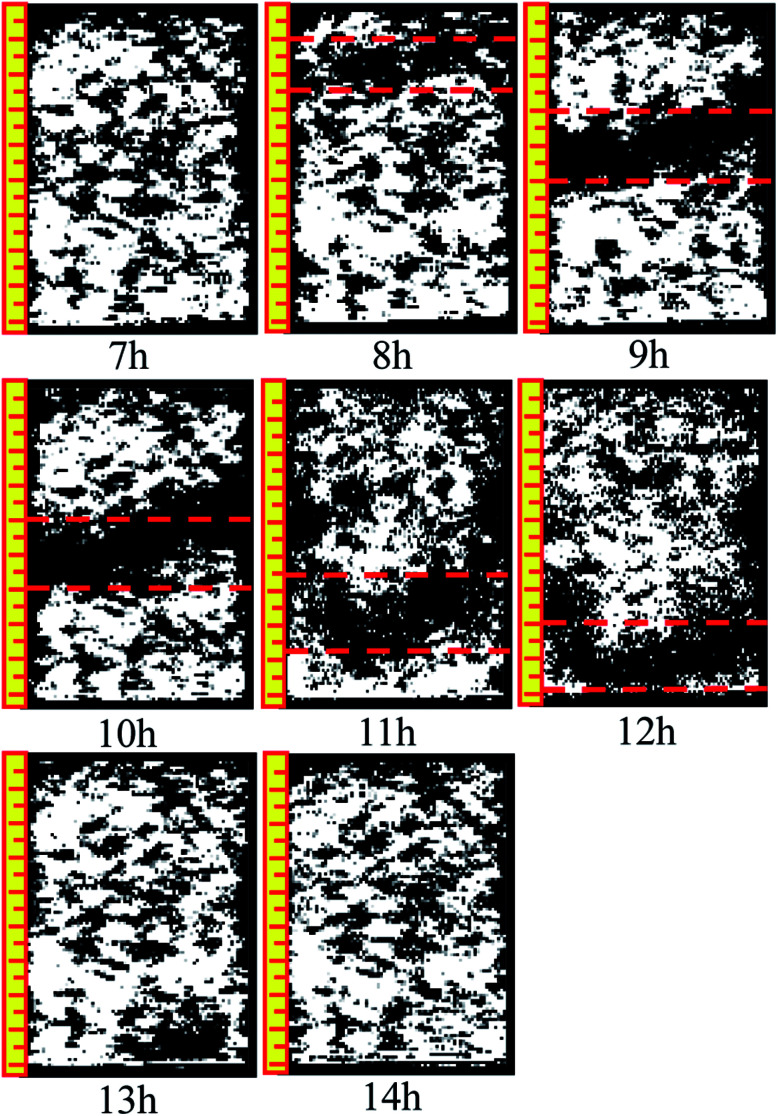
Phase of the (NH_4_)_2_SO_4_ solution (2.5%).

Fig. 10 and 11 are two-dimensional images of the cutting plane during the leaching process. Fig. 10 is the phase of pure H_2_O leaching, and Fig. 11 is the phase of the (NH_4_)_2_SO_4_ solution (2.5%). The bright colored area is the area of liquid molecules, which is the area of pores. The activity of the H_2_O molecules in the black area is extremely weak; thus, this area is considered a solid region.

An important change is shown in Fig. 11. The image starts to change when the leaching solution is replaced by the (NH_4_)_2_SO_4_ solution. In the 7^th^ hour, the bright area decreases slightly in the upper part, and the black area starts to increase. This area has a banding distribution in the upper part of the sample, but no clear change can be observed on the bottom of the sample compared with the image from the 6^th^ hour, indicating that (NH_4_)_2_SO_4_ and H_2_O would be effective in the specimen between the 6^th^ and 7^th^ hours. The chemical exchange area mainly appeared in the top of the specimen, which would change the particle structure; the pores with large apertures transform into pores with small and medium apertures, and the area of ion exchange tends to be more compact. The change becomes more obvious in Fig. 8: the black area grows increasingly large in the middle-upper part, which explains the rapid change in the pore structure under the effects of seepage and chemical reaction. The ion exchange reaction has been completed. Another clear change can be observed; namely, the bright area increases again in the middle-upper part, indicating that many pores have appeared in this area. The aperture is increasing, proving that the pore structure tends to be looser under the simple seepage effect. From the 9^th^ to 11^th^ hours, the area of the black stripe is moving downward and the bright area above the black area is recovering layer-by-layer. After 12 hours, the striped black area completely disappears, and the white area is widely distributed. According to Fig. 5, the ion exchange reaction has been completed at this point and only the seepage effect influences the micropore structure of the ore body.

The influence of pure H_2_O leaching on pore structure is the seepage effect while the influence of (NH_4_)_2_SO_4_ solution leaching on the micropore structure of ore body is ion exchange and seepage. The black area moving downward in Fig. 11 considerably differs from that in Fig. 10. Therefore, the micro structure of the ore will change under the hydro-chemical coupling effect. The ratio of large pores will increase and the micro structure will become compact. The trend that the layer moves down can be observed as well.

Many studies have found that 650 ppm REEs are only surface bound and are not part of the clay structure; therefore, their removal will not affect the clay structure. Based on simulated immersion tests, we found that pure water infiltration and ammonium sulfate infiltration methods yielded contradictory pore size trends. The pore distribution in different periods of sample infiltration and the black area the inversion image were obtained based on the NMR test. As the intense chemical exchange reaction ended, the black region disappeared, and the inverted image resembles the water infiltration image. Pure water infiltration, no matter how long the infiltration time, exhibited no black area. In summary, ammonium sulfate is associated with the appearance of black areas.

### 3.4. Permeability evolution during leaching

**Fig. 12:**
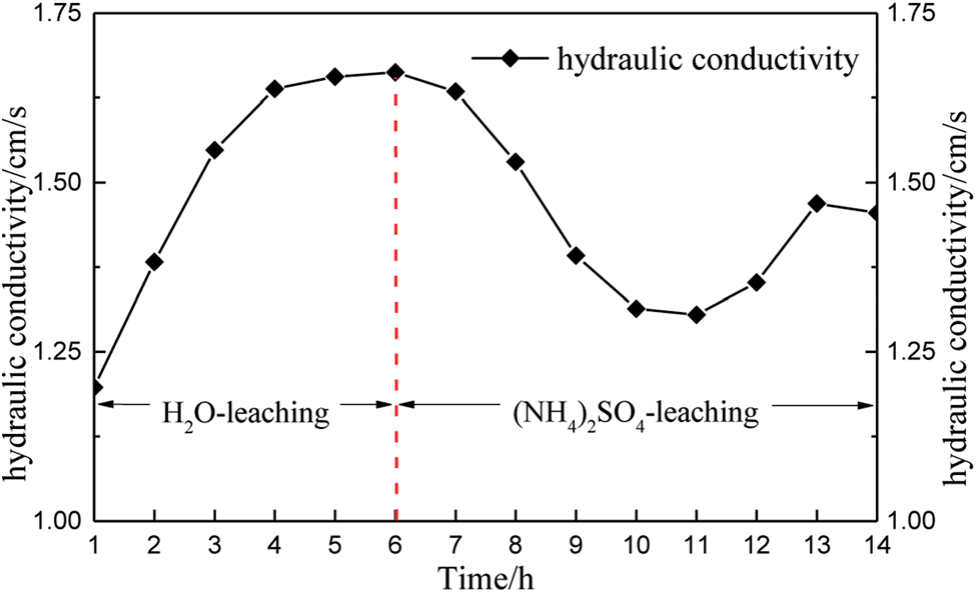
Hydraulic conductivity over time.

Soil permeability can be reflected by the hydraulic conductivity of the soil. Fig. 12 shows the specimen hydraulic conductivity over time.

Considering the changes in micropore structure and hydraulic conductivity (Fig. 12), the pore structure changed from small pores to large pores and the hydraulic conductivity increased under water seepage only, which indicated that water seepage facilitates solution seepage. Thereafter, the hydraulic conductivity tended to remain steady. When the solution changed to (NH_4_)_2_SO_4_, the pore structure changed from large pores to medium and small pores in 4 hours (6–10 h). The black belt shown in the pore structure image also indicates that the pore structure would compress under the effect of the chemical reaction. The result shows that the chemical replacement hinders solution seepage. Moreover, the permeability of rare earth ore is influenced more by chemical replacement than by solution seepage. However, the hydraulic conductivity increased again 11 hours later because the chemical replacement had completed at that point and the permeability of ore was influenced only by solution seepage. Therefore, the hydraulic conductivity increased.

Changes in the ionic strength, viscosity, and mineral components can cause this series of changes in pore size. Existing research has shown that variations in the ionic strength, viscosity and roles of other minerals are due to changes in the ion distribution of a solution. These changes in the ion distribution can cause the intensity of the electric field generated by the ions to change. Viscosity and ionic solubility are positively correlated and dynamically change. The exchange effect not only frees rare earth ions but also activates other ions. We can consider these effects a loss without any specific understanding of what factors play a leading role. In fact, it is necessary to study these factors in rare earth leaching because of the existence of exchange effects.

## 4. Conclusion

This paper conducted leaching experiments on ion-type rare earth ore bodies as well as a contrastive analysis of the leaching results of two different solutions. The following conclusions were obtained:

(1) The hydraulic conductivity of rare earth ore is influenced by solution seepage and chemical replacement, and the influence of chemical replacement is greater than that of solution seepage.

(2) The pore radius increases due to solution seepage, whereas it decreases due to particle recombination induced by chemical replacement.

(3) The hydro-chemical coupling effect will lead to variations in the pore structure and permeability of rare earth ore. The coupling effect is not conducive to solution seepage due to the dominant role of chemical replacement.

## Author contributions

X. J. W. and Y. L. Z. conceived and designed the experiments, analyzed the data, and wrote the paper. K. Z and W. Z. performed most of the experiments and analyzed the data.

## Conflicts of interest

The authors declare no conflicts of interest.

## Supplementary Material
